# THE TEMPORAL SEQUENCE OF FRAILTY, SOCIAL ISOLATION, AND LONELINESS IN OLDER ADULTS ACROSS 21 YEARS

**DOI:** 10.1093/geroni/igae098.0801

**Published:** 2024-12-31

**Authors:** Fereshteh Mehrabi, Mary Louise Pomeroy, Thomas Cudjoe, Emerald Jenkins, Elsa Dent, Emiel Hoogendijk

**Affiliations:** Concordia University, Montreal, Quebec, Canada; Johns Hopkins University, Baltimore, Maryland, United States; Johns Hopkins University School of Medicine, Baltimore, Maryland, United States; Johns Hopkins University, Baltimore, Maryland, United States; Torrens University, Adelaide, South Australia, Australia; Amsterdam University Medical Centers, Amsterdam, Noord-Holland, Netherlands

## Abstract

This study examined the temporal patterns of social isolation, loneliness, and frailty among a national sample of older adults across 21 years (1995–2016). We applied random intercept cross-lagged panel models to seven waves of the Longitudinal Aging Study Amsterdam (LASA). We included 2,302 Dutch older adults ages 55 and older (Mage= 72.6; SD = 8.6; 52.1% female). Frailty was measured using the Frailty Index. Loneliness was measured using the 11-item De Jong Gierveld loneliness scale. Social isolation was measured using a multi-domain 6-item scale. Levels of social isolation and loneliness were weakly correlated during any given wave as well as across waves. Each condition was highly stable from one time point (T) to the next. Cross-lagged effects showed that frailty in T2 to T5 significantly predicted social isolation in the following waves (T3 to T6). For example, higher levels of frailty at T4 predicted higher levels of social isolation at T5 (β = 0.23, SD = 0.05, p < 0.001). Likewise, frailty in T2 to T6 significantly predicted loneliness in the following waves (T3 to T7). For example, higher levels of frailty at T5 predicted higher levels of loneliness at T6 (β = 0.33, SD = 0.06, p < 0.001). The reverse sequence (i.e., social isolation or loneliness predicting future frailty) was less common and exhibited a weaker relationship. Frailty may have important consequences for adverse psychosocial outcomes, including social isolation and loneliness. Public health policies should prioritize interventions that bolster social connection among pre-frail and frail older adults.

